# P-970. Impressions of the Pediatric Infectious Diseases Subspecialty by Pediatric Resident Physicians and Medical Students Following a Peds ID Elective Rotation

**DOI:** 10.1093/ofid/ofae631.1160

**Published:** 2025-01-29

**Authors:** Michael E Watson

**Affiliations:** University of Michigan, Ann Arbor, Michigan

## Abstract

**Background:**

The subspecialty of Pediatric Infectious Diseases continues to struggle with a low number of applicants seeking training relative to the number of fellowship programs and open training positions. We explored the impressions that medical students and pediatric residents gathered about the subspecialty after completion of a Peds ID elective as a Quality Improvement initiative.

**Methods:**

Resident physicians and medical students at the University of Michigan, from July 2022 to present, were surveyed after their Peds ID elective experience. Participation was voluntary and only anonymous responses in aggregate form were reported.

**Results:**

The survey response rate was 56.3% (27 of 48 learners invited; 79% resident physicians and 21% medical students). 100% of learners reported a high educational value in participating in a Peds ID elective. Despite only 7.4% of learners considering Peds ID as a career before the elective, 18.5% of learners reported considering Peds ID fellowship training after their elective experience. Respondents indicated that 1) expertise in the subject matter of infectious diseases, 2) a mixture of inpatient and outpatient clinical experiences, and 3) opportunities to participate in Infection Prevention and Public/Global Health, were the top three most attractive aspects of the Peds ID subspecialty. In contrast, respondents indicated that 1) salary and compensation, 2) antimicrobial stewardship responsibilities, and 3) patient volume and workload, were the three least attractive aspects of the Peds ID subspecialty.

**Conclusion:**

Peds ID remains a popular elective among learners who praise the educational content and teaching. Relatively low compensation, a heavy workload, and displeasure with antimicrobial stewardship duties were some of the main negative impressions by learners. Despite these impressions, participation in a Peds ID elective did lead to over twice as many learners considering Peds ID for their future careers. A mandatory Peds ID experience should be considered for the educational benefit of all learners and may improve fellowship recruitment. We identified issues for our subspecialty to address through advocacy to improve working conditions and compensation, thereby helping train and sustain an appropriate Peds ID workforce for the future.

**Disclosures:**

**All Authors**: No reported disclosures

